# Scriptaid overcomes hypoxia-induced cisplatin resistance in both wild-type and mutant p53 lung cancer cells

**DOI:** 10.18632/oncotarget.12378

**Published:** 2016-09-30

**Authors:** Shrikant Pradhan, Divyank Mahajan, Prabhjot Kaur, Namita Pandey, Chandresh Sharma, Tapasya Srivastava

**Affiliations:** ^1^ Department of Genetics, University of Delhi South Campus, New Delhi, India; ^2^ Centre for Bio-design and Diagnostics, Translational Health Science and Technology Institute, Faridabad, Haryana, India

**Keywords:** lung cancer, cisplatin, scriptaid, hypoxia, metastasis

## Abstract

Non-small cell lung cancer (NSCLC), comprising 85% of lung cancer cases, has been associated with resistance to chemo/radiotherapy. The hypoxic tumor micro-environment, where insufficient vasculature results in poor drug penetrance and sub-optimal chemotherapy in the tumor interiors contributes heavily to this resistance. Additionally, epigenetic changes in tumorigenic cells also change their response to different forms of therapy. In our study, we have investigated the effectiveness of a combination of cisplatin with scriptaid [a pan-Histone Deacetylase inhibitor (HDACi)] in a model that mimics the tumor microenvironment of hypoxia and sub-lethal chemotherapy. Scriptaid synergistically increases the efficacy of cisplatin in normoxia as well as hypoxia, accompanied with reduced metastasis and enhanced DNA damage. Addition of scriptaid also overcomes the cisplatin resistance exhibited in lung cancer cells with stabilized hypoxia inducible factor 1 (HIF1)-α (mutant) and mutant p53. Molecular studies showed that the combination treatment increased apoptotic cell death in both normoxia and hypoxia with a dual role of p38MAPK. Together, our results suggest that the combination of low dose cisplatin and scriptaid is cytotoxic to NSCLC lines, can overcome hypoxia induced resistance and mutant p53- induced instability often associated with this cancer, and has the potential to be an effective therapeutic modality.

## INTRODUCTION

Lung cancer is a malignancy of the respiratory epithelium and the leading cause of cancer related deaths worldwide. Of the two types of lung cancer, small cell lung cancer (SCLC) and non-small cell lung cancer (NSCLC), the latter comprises about 85% of the total cases [1]. Among the different genetic abnormalities associated with lung cancer, mutations in *p53*, *KRAS*, *EGFR* and *LKB1* are the most common. Mutations in p53 gene occur early in cancer development and are maintained throughout the advanced stages of tumor development [2, 3]. With an overall survival rate of five years, the treatment outcome of NSCLC still remains poor. However, the management of locally advanced NSCLC has progressed significantly with the use of combined therapeutic interventions [4]. Standard treatment for advanced stages of NSCLC is surgery followed by platinated drug-based adjuvant therapy. Although cisplatin is a drug of choice for NSCLC treatment [5, 6], patients are known to acquire drug resistance.

Overcoming cisplatin-resistance, therefore, is a crucial factor for developing anticancer therapy [7]. The hypoxic tumor micro-environment is one of the key players in acquired chemo/radio resistance mainly due to poor penetrance. The resulting low doses in tumor interiors leads to metabolic and genetic changes in a hypoxic cell favoring cell proliferation, angiogenesis and metastasis [8]. We have shown previously, that low dose cisplatin induces genomic instability in hypoxic glioma cells [9]. Hypoxia inducible factor1α (HIF1α), identified by Wang and Semenza, is the oxygen sensor which activates a host of hypoxia inducible genes [10]. Deletion of *HIF1α* in a mouse mammary tumor virus (MMTV) promoter-driven polyoma middle T antigen mouse model of breast cancer showed reduction in growth of the primary tumors [11]. In contrast, *HIF1α* deletion in a KRAS-driven mouse model of lung cancer had negligible effect on tumor burden and progression, whereas a deletion in its isoform, *HIF2α* increased tumor growth and progression [12]. In renal cell carcinoma, stabilization of HIF1α reduced tumor progression, while overexpression of *HIF2α* resulted in increased tumorigenesis [13, 14]. Thus, the role of HIF isoforms is complex and differs with tumor and stromal cell types.

Overcoming this chemo-resistance with the help of specific inhibitors and modulators has had moderate success. For example, initial trials of tirapazamine (TPZ) in combination with chemo/radiotherapy showed promising results in NSCLC, cervical and head and neck cancers [15–18] however, subsequent phase III trials failed to demonstrate its effectiveness [19, 20].

Alterations in epigenetic marks-histone acetylation and methylation of gene promoters- are also known to contribute to the onset and progression of various types of cancers [21]. Hypoxia has been shown to induce epigenetic changes in the cancer genome by us [22] and others [23–25]. In hypoxic conditions, histone deacetylases interact with HIF1α to regulate the expression of many genes. While interaction of HIF1α with HDAC1 down-regulates HIF-targets such as RECK and Reptin [26, 27], recruitment of HDAC4, HDAC5 and HDAC7 increases the expression of HIF1α-regulated genes [28].

We have investigated the ability of scriptaid, a pan HDAC inhibitor (HDACi) in sensitizing lung cancer cells to low dose cisplatin. A low dose cisplatin is reflective of what the hypoxic interiors of lung cancer are likely to receive and what a patient needs, to minimize side-effects. Considering the well-established role of hypoxia and HIF1 in regulating key cellular and molecular pathways in cancer progression, it seems relevant to investigate therapeutic molecules and regimes in *in vitro* hypoxic conditions. We have determined whether this combination treatment can overcome cisplatin-resistance in hypoxia and have investigated the underlying mechanism of cell death. We have also studied the effectiveness of this combination therapy in the p53 dominant negative lung cancer cells, hence addressing the prevalence of a significant percent of NSCLC. Lastly, we have studied combination therapy in mutant HIF1α overexpressing genetic background to address the molecular mechanism in more detail.

## RESULTS

### Cisplatin and scriptaid induce dose dependent cytotoxicity in lung cancer cells

**Figure 1:**
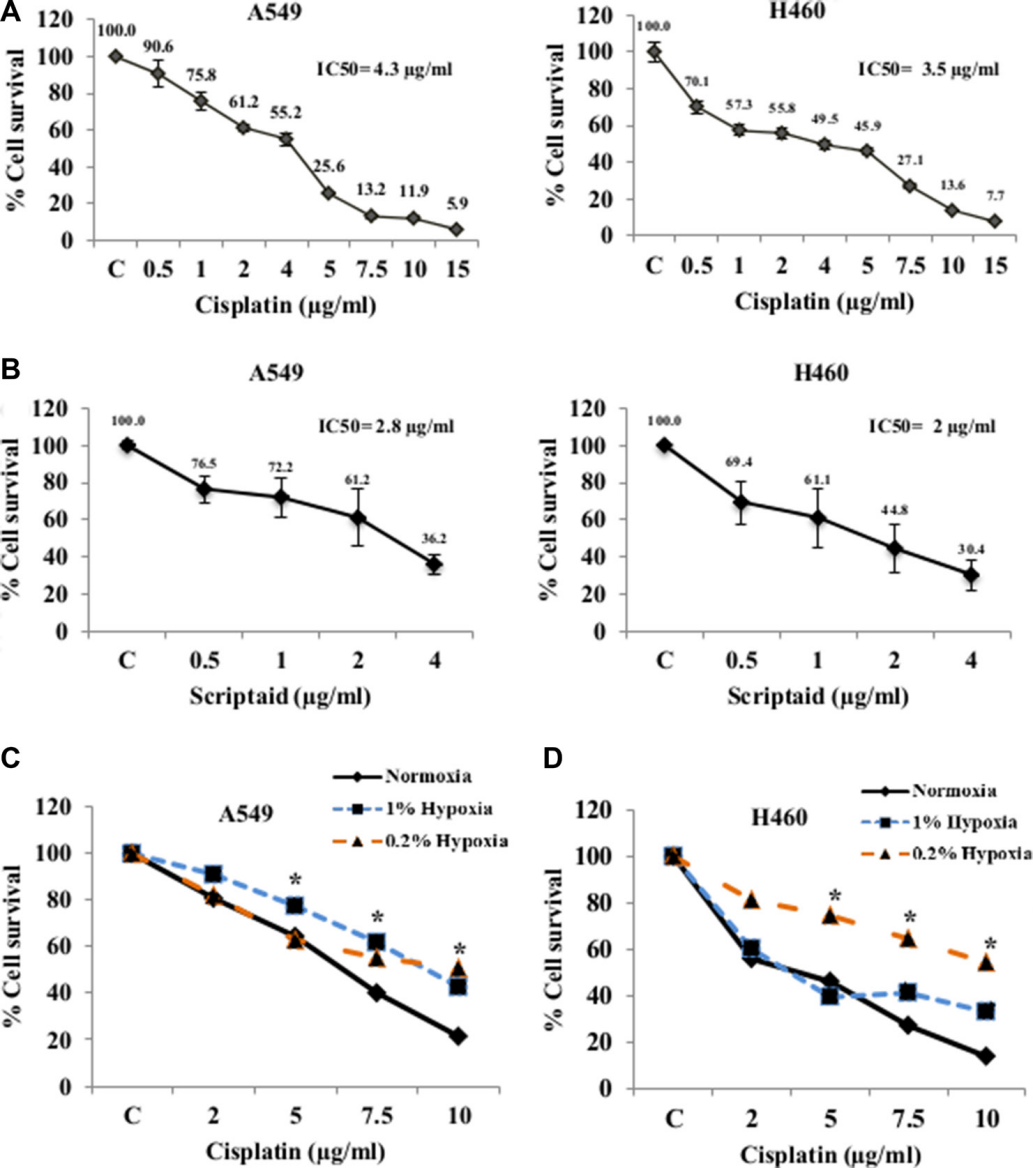
Cytotoxicity assay for Cisplatin and Scriptaid on lung cancer cells MTT assay of A549 and H460 cells for cytotoxicity to cisplatin (**A**) and scriptaid (**B**). The IC50 values of A549 and H460 cells for cisplatin was 4.3 and 3.5 μg/ml and that for scriptaid was 2.8 and 2 μg/ml respectively. MTT assay for cisplatin in A549 (**C**) and H460 (**D**) cells showing resistance to the drug in hypoxic conditions (1% and 0.2%) compared to that in normoxia. C= untreated control. * indicates *p* < 0.05.

Lung cancer cell lines, A549 and H460 showed dose dependent cell cytotoxicity with both cisplatin (Figure 1A) and scriptaid (Figure 1B) in normoxia. The IC50 concentration of cisplatin for A549and H460 was 4.3 μg/ml and 3.5 μg/ml, respectively. The IC50 concentration of scriptaid for A549 and H460 was determined at 2.8 μg/ml and 2 μg/ml, respectively. To confirm the activity of scriptaid, A549 cells were treated with three different concentrations for 24 hrs and western blot analysis performed for the acetylated histone H4. A concentration-dependent increase in the expression of acetylated histone H4 was observed in response to scriptaid (Supplementary Figure S1A).

### Lung cancer cells showed cisplatin resistance in hypoxia

Solid tumors are known to harbor heterogeneous hypoxic regions in the interiors. We have assessed cytotoxicity to cisplatin treatment in cells cultured in two grades of hypoxia-1% O2 (1% hypoxia) and 0.2% O2 (0.2% hypoxia). As expected, they showed variable response to cytotoxicity. In 1% hypoxia, A549 cells showed significant resistance to cisplatin compared to that in normoxia for all four doses. But in near-anoxic condition of 0.2% hypoxia, A549 cells exhibited resistance only at higher doses (Figure 1C). On the contrary, H460 cells showed resistance to all doses of cisplatin in 0.2% hypoxia whereas in conditions of 1% hypoxia the cells showed resistance at higher doses of cisplatin but not in lower and median doses (Figure 1D). Induction and stabilization of HIF1α, was confirmed by western blotting in A549 cells at both doses of hypoxia (Figure S2A). Overexpression of HIF1α-inducible genes- *GLUT1* and *CAIX* validated the conditions of *in vitro* hypoxia (Figure S2B). Cell cycle analysis using flow cytometry showed a G2-M and S-phase arrest at lower doses of cisplatin and a dose-dependent increase in cell death in normoxic condition. However, in hypoxic conditions, cell cycle arrest was averted and the amount of cell death was relatively lower. The G0-G1 population was significantly higher in hypoxic conditions than that observed in normoxia (Supplementary Figure S2C).

### Scriptaid acts synergistically with cisplatin and overcomes hypoxia-induced cisplatin resistance

**Figure 2:**
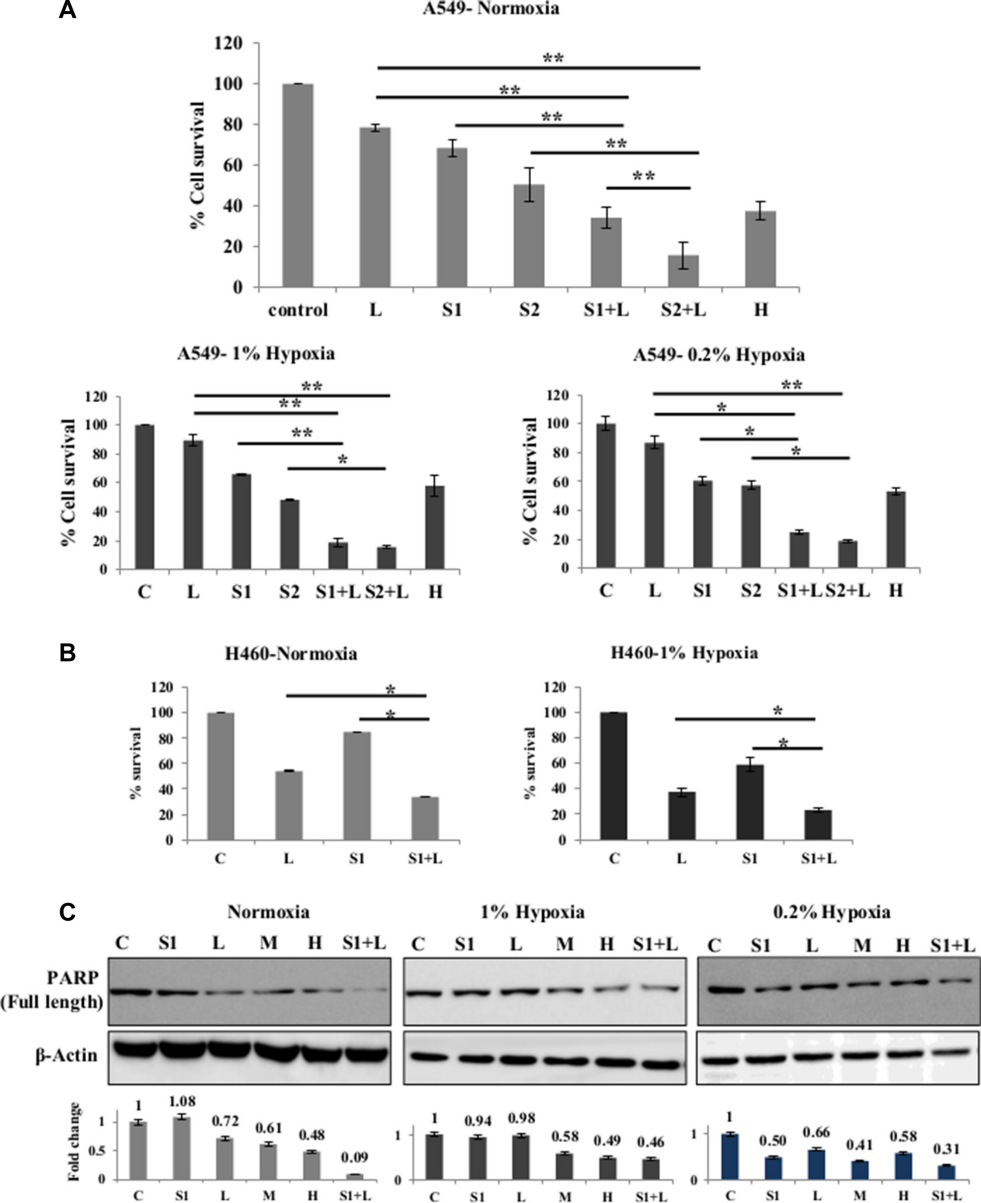
Synergistic effect of combination treatment (**A**) Combination of cisplatin and scriptaid at sub-lethal doses showing significantly enhanced cytotoxicity than either of single agent treatment and high dose cisplatin. The combination remained effective in both condition of hypoxia- 1% and 0.2%. (**B**) MTT assay of H460 cells shows that the combination treatment was significantly more effective than either of the single agent in both normoxic and hypoxic conditions. (**C**) Cleavage of PARP as seen by western blot analysis as a marker for cell death. High dose cisplatin and combination treatments resulted in greater amount of cell death in all conditions. Graphs represent the fold change in expression of cleaved PARP, normalized with the loading control- β-actin. C= untreated control, L=2μg/ml cisplatin, M= 5μg/ml cisplatin H= 7.5μg/ml cisplatin, S1= 1μg/ml scriptaid, S2= 2μg/ml scriptaid. * indicates *p* < 0.05, ** indicates *p* < 0.005.

One low dose of cisplatin (2 μg/ml) and two doses of scriptaid (1 μg/ml and 2 μg/ml) were selected for studying cytotoxicity with combination therapy in A549 and H460 cells. Sequential addition of drugs- scriptaid treatment for 2–4 hrs followed by cisplatin treatment- did not result in elevated cytotoxicity (data not shown). Simultaneous addition of both the drugs showed promising results. The combinations used- 2 μg/ml cisplatin+1 μg/ml scriptaid and 2 μg/ml cisplatin+2 μg/ml scriptaid-synergistically improved the efficacy of low dose cisplatin in normoxic conditions (*p* < 0.005) in A549 (Figure 2A). Synergism was calculated as described by Kern *et al.* (1998), where, an R index of >1.5 signifies synergism. Further, the combination proved to be effective in overcoming cisplatin resistance in both conditions of hypoxia- 1% and 0.2 (*p* < 0.005). The R index of both combinations were > 1.5 in 1% hypoxia and 0.2% hypoxia (Figure 2A). DAPI staining of A549 cells showed an overall higher number of apoptotic population in combination treated samples compared to that observed in mono therapy (Supplementary Figure S1B). It was observed that scriptaid did not influence the expression of HIF1α under hypoxia (Supplementary Figure S4A). Although single agent cisplatin and scriptaid were less effective in hypoxic conditions, the combination treatment leads to an increase in apoptosis in these cells. Poly-ADP ribose polymerase (PARP) expression further supports cytotoxicity data, where the combination therapy synergistically improves the toxicity of low dose cisplatin in all three conditions- normoxia, 1% hypoxia and 0.2% hypoxia (Figure 2B). The combination treatment was also effective in H460 cells in both normoxic and hypoxic conditions (Figure 2C).

### Combination therapy inhibits migratory behavior of A549 cells

**Figure 3:**
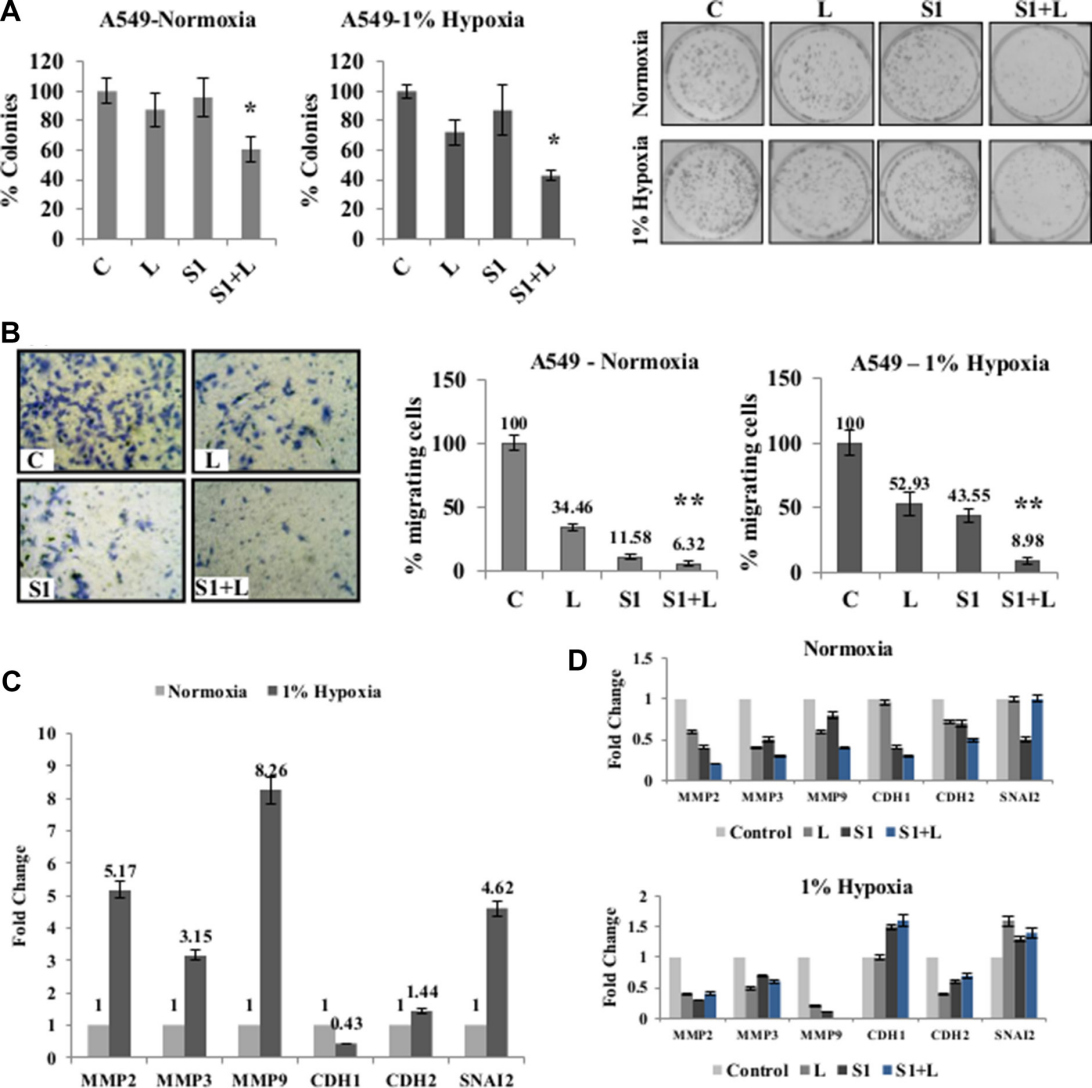
Combination treatment impairs clonogenic and migratory potential of cancer cells (**A**) Clonogenic assay shows a significant inhibition of colony formation in doublet treated samples compared to single agent cisplatin and scriptaid in normoxia as well as 1% hypoxia. (**B**) Representative picture of migration assay in normoxia; Migration assay using Boyden chambers shows a significant inhibition of cell migration in combination treatment compared to single agent treatment in both normoxic and hypoxic conditions. (**C**) Real-time PCR analysis of EMT markers showed an up-regulation of mesenchymal markers and down-regulation of epithelial marker under hypoxic condition when normalized against normoxia. (**D**) Real-time PCR of EMT markers in response to mono and combination treatment in normoxic and hypoxic conditions shows down-regulation of mesenchymal markers and up-regulation of epithelial marker in hypoxic condition. C= control, L=2μg/ml cisplatin, S1= 1μg/ml scriptaid. MMP= matrix metalloprotinase; CDH1= cadherin1/E-cadherin; CDH2= cadherin1/N-cadherin; SNAI2= Snail2. * indicates *p* < 0.05, ** indicates *p* < 0.005.

For further analysis, a combination of 2 μg/ml cisplatin+1 μg/ml scriptaid was used for all *in vitro* studies. Clonogenic assay showed that the combination treatment resulted in significantly fewer colonies compared to cisplatin and scriptaid monotherapy (*p* < 0.05) (Figure 3A) and inhibited wound healing in both normoxia and hypoxia (Supplementary Figure S3). In hypoxic conditions, single agent treatment did not inhibit cell migration to the same extent as that in normoxia. However, the doublet treatment significantly inhibits cell migration in both conditions (Figure 3B). Enhanced migratory behavior of lung cancer cells in hypoxic conditions can be attributed to the overexpression of mesenchymal markers viz. *MMP-2*, *3*, *9*, *CDH2* and *SNAI2*, together with the down-regulation of *CDH1*- an epithelial marker (Figure 3C). Further analysis of the EMT markers in response to treatment shows that the combination treatment down-regulated the expression of matrix metalloproteases (MMPs). The epithelial marker, *CDH1*, was up-regulated in response to combination treatment in hypoxia (Figure 3D). Thus, the combination treatment inhibits cell migration by repressing mesenchymal genes.

### Combination therapy leads to apoptotic cell death in A549 cells

**Figure 4:**
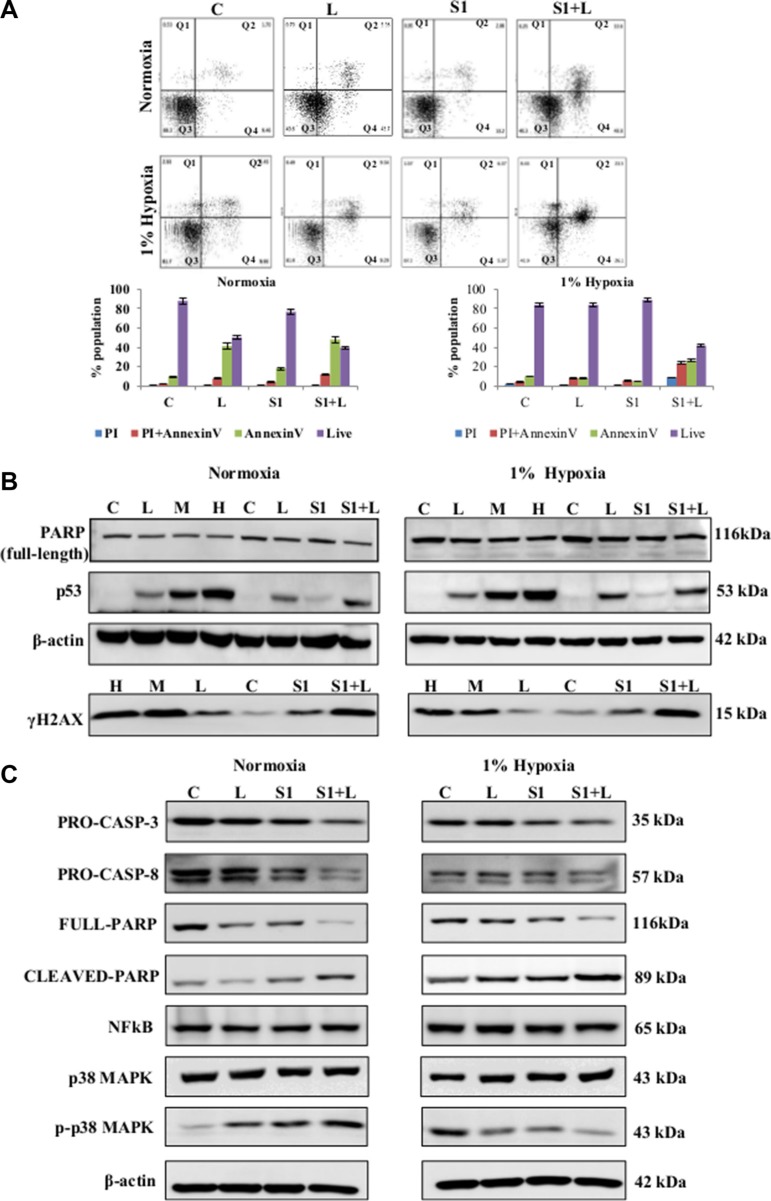
Molecular analysis of cell death and cell signaling markers (**A**) AnnexinV-PI dual staining and flow cytometry analysis of lung cancer cells showed elevated levels of apoptotic cell death in combination treatment in normoxia as well as hypoxia. (**B**) Western blot analysis for DNA damage and cell death markers shows a synergistic improvement in combination treatment compared to low dose single agent treatment. (**C**) Western blot analysis shows the activation of caspase-mediated apoptosis in response to combination treatment in both normoxic and hypoxic conditions. Q1=necrotic population, Q2=late phase apoptosis, Q3=live cells, Q4= early phase apoptosis. C= control, L=2μg/ml cisplatin M= 5μg/ml cisplatin, S1= 1μg/ml scriptaid.

The combination therapy induced apoptotic cell death in both normoxic and hypoxic conditions as determined by AnnexinV labeling (Figure 4A). Single agent cisplatin and scriptaid did not induce apoptosis. However, majority of the cell population in combination treated samples were in early and late phase apoptosis. In combination treated samples of both normoxia and hypoxia, enhanced expression of p53 and γ-H2AX was observed (Figure 4B). Cleavage of caspase-3 and caspase-8 indicates that the combination treatment activates the extrinsic pathway of apoptotic cell death (Figure 4C). NFκB expression remained unaffected by mono and combination treatment in both conditions. The expression of phosphorylated-p38MAPK (p-p38MAPK) was differentially regulated compared to total p38MAPK. The increased accumulation of p-p38MAPK in treated samples indicates its role in apoptosis in normoxic condition while, the over-expressed p-p38MAPK in untreated hypoxic conditions is significantly reduced in treated samples (Figure 4C).

### Scriptaid sensitizes cisplatin-resistant HIF1α-stable mutant cells to apoptotic cell death

**Figure 5:**
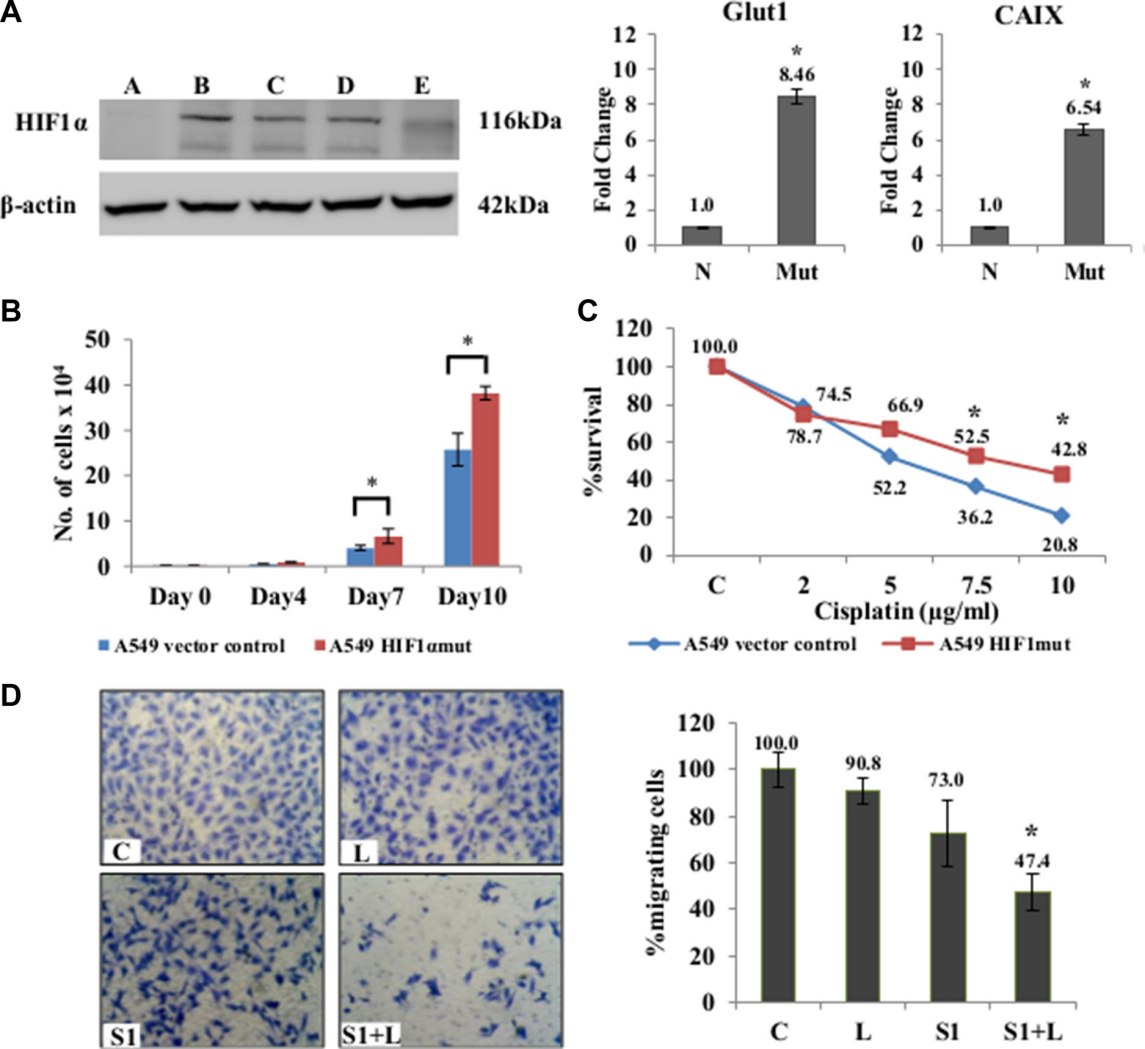
Effects of combination treatment in mutant-HIF1α expressing A549 cells (**A**) Confirmation of stable clones was done by western blot analysis using HIF1α specific antibody. Clone B was selected for further experiments. qRT-PCR showed an up-regulation of of HIF1-inducible genes (Glut1 and CAIX) in A549/HIF1 α mut cells. The A549/HIF1α mut cells showed a higher rate of proliferation (**B**) and resistance to cisplatin (**C**) compared to A549 cells with wt-HIF1α. (**D**) Migration assay using Boyden chambers showed a significant inhibition of migration in A549/HIF1mut cells in response to combination treatment than that seen in monotherapy. A= empty vector control; B, C, D, E= different selected clones. C= control, L=2μg/ml cisplatin, S1= 1μg/ml scriptaid. * indicates *p* < 0.05.

**Figure 6:**
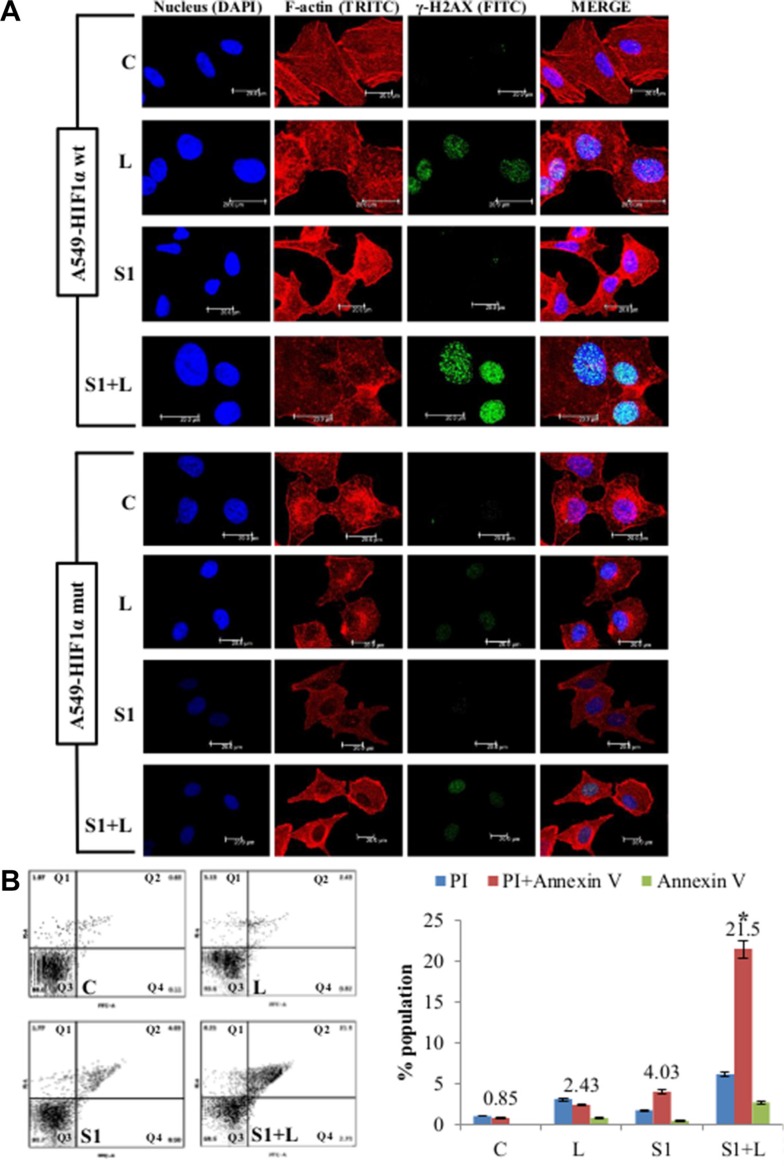
Cell death and DNA damage in response to treatment in A549/HIF1α mut cells (**A**) Confocal images of A549/wt-HIF1α and A549/HIF1α mut shows an elevated level of DNA damage in combination than single agent treatment. Increased accumulation of γ-H2AX indicates DNA damage. Nucleus= Blue (DAPI), F-actin= Red (TRITC), γ-H2AX= Green (FITC). (**B**) AnnexinV-PI dual staining and flow cytometry analysis shows significantly higher apoptosis in combination treatment than low dose cisplatin and scriptaid. Q1=necrotic population, Q2=late phase apoptosis, Q3=live cells, Q4= early phase apoptosis. C= control, L=2μg/ml cisplatin, S1= 1μg/ml scriptaid. * indicates *p* < 0.05.

One of the key players in regulating hypoxia-induced cisplatin-resistance is the HIF1α. We generated a HIF1α overexpressing stable mutant (HA-HIF1alpha P402A/P564A-pcDNA3 vector) cell line to study the contribution of HIF1α to our results on hypoxia. Stably transfected A549 cells showed elevated HIF1α expression even under normal oxygen concentration (Figure 5A). Up-regulation of *GLUT1* (about 8 folds) and *CAIX* (about 6 folds), the two HIF1α-inducible genes, in A549-HIF1α mut cell line compared to the vector control cell line (Figure 5B) confirmed the effectiveness of HIF1α overexpression. The mutant cell line also had a proliferative advantage over the vector control A549 cells (Figure 5C). They showed resistance to cisplatin induced cytotoxicity compared to A549 cells with wt-HIF1α (Figure 5D) similar to our results in hypoxia. The combination treatment also significantly reduced cell migration (*p* < 0.05) in the mutant cell line compared to single agent treatment (Figure 5E). Immunofluorescence staining using γ-H2AX antibody showed that the mutant cell line was more resistant to single agent cisplatin and scriptaid than the wild type counterpart, while the combination therapy resulted in an increased amount of DNA damage (Figure 6A). The corrected total fluorescence (CTCF) was significantly higher in combination treatment than single agent treatment (Supplementary Figure S4B). Lastly, annexinV/PI dual staining and flow cytometry analysis shows the enhanced cytotoxicity via apoptosis of combination treatment in a HIF1α over-expressing mutant background (Figure 6B) similar to that in physiological hypoxia.

### Cisplatin resistant A549/p53DN cells undergo apoptotic cell death in presence of scriptaid

**Figure 7:**
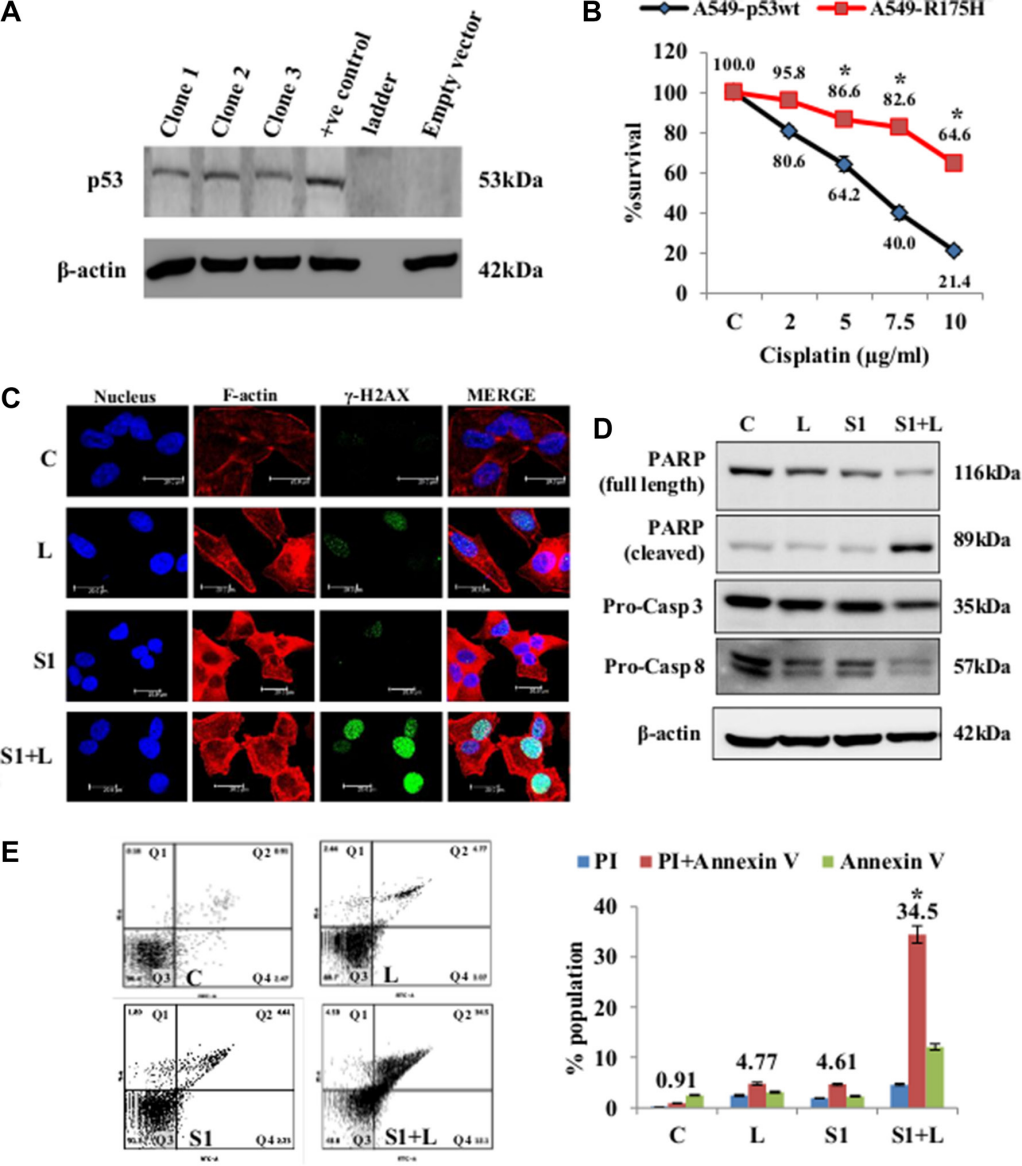
Cell death and DNA damage in response to treatment in A549/p53 dominant-negative cells (**A**) Confirmation of stable clones was done by western blot analysis using p53 specific antibody. (**B**) MTT assay for cisplatin shows that the mutant cell line is resistant to the drug as compared p54-wt A549 cells. (**C**) Confocal images of A549/p53 dominant negative cells shows an elevated level of DNA damage in combination than single agent treatment. Increased accumulation of γ-H2AX indicates DNA damage. Nucleus= Blue (DAPI), F-actin= Red (TRITC), γ-H2AX= Green (FITC). (**D**) Western blot analysis shows a caspase mediated cell death in A549/p53 mut cells in response to treatment. (**E**) AnnexinV-PI dual staining and flow cytometry analysis shows significantly higher apoptosis in combination treatment than low dose cisplatin and scriptaid. Q1=necrotic population, Q2=late phase apoptosis, Q3=live cells, Q4= early phase apoptosis. C= control, L=2μg/ml cisplatin, S1= 1μg/ml scriptaid. * indicates *p* < 0.05.

Mutations in p53 are common in cancer, with R175H position mutation being the most common. We have generated a stable p53-R175H-dominant negative (DN) mutant in A549 cell line to answer two questions- (a) whether this mutation affects the sensitivity of cancer cells to cisplatin and (b) can scriptaid improve the efficacy of cisplatin in such a mutant background. The pCMV-Neo-BamR175H vector codes for a mutant p53 gene which dimerizes with cellular wt-p53 and renders it non-functional. The stable mutant cell line was generated and confirmed by western blot analysis using p53-specific monoclonal antibody (Figure 7A). Cytotoxicity assays for cisplatin showed that the mutant cell line was significantly resistant to all doses of cisplatin compared to the wild-type control (Figure 7B). MTT assay of A549-R175H in both normoxic and hypoxic conditions shows a synergistic effect of combination treatment (Figure S4C). In normoxic condition, A549-R175H cells were less sensitive to single agent cisplatin and scriptaid, while the combination therapy lead to an increased accumulation of γ-H2AX indicating greater amount of DNA damage in these samples (Figure 7C). Protein expression of PARP, caspase-3 and caspase-8 shows that the combination treatment leads to apoptotic cell death in A549-R175H cells whereas single agent treatment proved ineffective (Figure 7D). Further proof of apoptotic cell death was provided by annexinV/PI staining and flow cytometry analysis (Figure 7E). The combination therapy led to significantly greater amount of cell death compared to monotherapy with either cisplatin or scriptaid. Thus, combination therapy with low dose cisplatin and scriptaid can also overcome cisplatin-resistance which results from a dominant-negative mutation of p53 in lung cancer cells. Molecular analysis of apoptosis markers in hypoxia shows an activation of caspase-8, while caspase-3 was not significantly affected by the combination treatment (Supplementary Figure S4C).

## DISCUSSION

The hypoxic tumor microenvironment is a fertile bed for chemo-resistance, genetic alterations and changes in cellular phenotype. Low drug perfusion in hypoxic interior of tumors changes the cytotoxicity and the associated signaling networks. Cisplatin is a commonly used drug for the treatment of non-small cell lung cancer and patients often develop resistance to cisplatin over the course of therapy and multiple mechanisms have been attributed to the resistance, including hypoxia [31, 32]. On-going research efforts aim to overcome this chemo-resistance, however, the outcomes have been limited. In our study we have addressed this hurdle of hypoxia-induced cisplatin resistance in non-small cell lung cancer cells *in vitro*, by using a combination of low dose cisplatin and scriptaid.

We have noted dose-dependent cytotoxicity of cisplatin and scriptaid on two lung cancer cell lines- A549 and H460. Both cells lines showed resistance to cisplatin in the two grades of hypoxia studied, albeit at variable doses. Thus, the heterogeneity of tumor hypoxia elicits varied responses depending on the cells of origin. Similar to other reports [33, 34], cisplatin induced an S/G_2_-M phase arrest coupled with reduced G_0_-G_1_ cell population. In hypoxic conditions, however, lung cancer cells overcame S/G2-M phase arrest along with an increase in G0-G1 population, a characteristic feature of hypoxia [35].

HDAC inhibitors such as TSA (trichostatin A) and panobinostat have shown potential in overcoming drug resistance in cancer therapy [36, 37]. While the use of HDAC inhibitors such as TSA and SAHA (suberoylanilide hydroxamic acid) in cancer therapy has been well studied, investigations on Scriptaid- a novel pan-HDAC inhibitor- are less reported. We selected sub-lethal doses of cisplatin and scriptaid for combination treatment to mimic conditions likely to occur in hypoxic tumor interiors. In all three conditions- Normoxia, 1% hypoxia and 0.2% hypoxia- the combination proved to be significantly more effective than monotherapy. The synergistic effect of combination was calculated by the method described by Kern et al. [38]. The combination was particularly more effective in hypoxic conditions and led to more cell death than single agent cisplatin. Low doses of either cisplatin or scriptaid did not lead to significant DNA damage as a single agent, while the combination elicits high levels of DNA damage in both normoxia and hypoxia as observed by morphology and flow cytometry analysis using annexinV/PI dual staining.

Cisplatin is known to induce p53 and lead to the activation of mitochondrial apoptosis pathway [39]. We observed enhanced p53 activation with increasing dose of cisplatin. p53 expression in combination treatment was not similar to high dose cisplatin. Thus, augmentation of apoptosis upon doublet treatment may also involve other pathways apart from the p53-induced apoptosis. Cleavage of pro-caspase3 and pro-caspase 8 indicates that the extrinsic pathway of apoptosis is active in doublet treated samples. Our results indicate that p-p38MAPK plays different roles in survival and cell death in response to the micro-environment. p38MAPK has shown variable characteristic in many studies, of promoting cell death [40, 41] and enhancing survival, depending on the conditions and cell-type [42]. Expression of p-p38MAPK appears to play an important role in apoptosis in normoxic condition, while it may provide cell survival signals in hypoxic micro-environment. NFκB is a major downstream effector of hypoxia response [43, 44] and an important player in the immune response to cancer. While, NFκB expression was elevated in hypoxia, its expression remained unaffected in response to treatment in both conditions. Tumor hypoxia has been shown to be associated with necrotic cell death [45] and we observed marginal necrosis in cells exposed to hypoxia. That the combination treatment proved to be more toxic and resulted in higher apoptosis than single agent cisplatin or scriptaid, even in hypoxia, is an extremely relevant and significant finding. Single agent treatment retards cell migration and the combination furthers inhibits migratory properties of cancer cells as observed by functional studies and molecular analysis of EMT markers.

To determine whether HIF1α is the major effector molecule of our observations on cisplatin-resistance in hypoxia, we generated stable A549 cells expressing a mutant form of HIF1α, which is stabilized under normoxia, while maintaining the wild-type functions. A549/HIF1α mut cells were significantly more proliferative than cells with wild-type HIF1α. Over-expression of HIF1α has been associated with enhanced cell migration of cancer cell [46, 47]. The A549/HIF1α mut cells were highly migratory, and similar to cells exposed to hypoxia, responded weakly to single agent treatment. The combination treatment significantly enhanced DNA damage and apoptotic cell death in A549/HIF1α mut cells apart from inhibiting cell migration. Thus, we conclude that cisplatin-resistance of lung cancer cells in hypoxic micro-environment is mostly regulated by HIF1α and the combination therapy overcomes this resistance.

In order to address the role of major genetic mutation in NSCLC, we generated a dominant negative p53 expressing stable A549 cells- A549-R175H p53 mutant, to determine the effectiveness of the combination therapy in this genetic background. p53 mutation is a common phenomenon in lung cancer and linked to decreased survival in patients receiving cisplatin-based chemotherapy [48], TP53 mutational spectrum in human cancers has shown the R175H mutation occurs most frequently with an overall frequency of 4.6% [49]. The A549-R175H cells were significantly resistant to all doses of cisplatin compared to A549 cells with wt-p53. Immunostaining with γ-H2AX showed that single agent cisplatin and scriptaid did not result in significant DNA damage in A549/R175H cells. The combination therapy, however, led to synergistic enhancement in DNA damage. Using annexinV/PI dual staining and western blot analysis with cell death markers, we have shown that the combination therapy is effective in a p53-dominant negative genetic background and also overcomes cisplatin resistance in lung cancer cells.

In our study we have determined the effectiveness of low doses scriptaid and cisplatin combination therapy in the heterogeneous tumor microenvironment. Apart from the pre-dominant *EGFR* and *RAS* mutations that occur in most NSCLC, significant population of patients acquire p53 (R175H) mutations. The combination dose was, hence, tested for cytotoxicity *in vitro* on the genotype observed in two main groups of activating mutations found in NSCLC patients, the *EGFR-LKB1-RAS* (A549 cell line) and *EGFR-LKB1-RAS-p53* (A549-R175H p53 mutant cell line), in both normoxia and hypoxia.

Presently, the success rate of drugs from pre-clinical phase to clinical trials remains poor. The combination of low dose cisplatin and scriptaid is equally effective in hypoxia, and possibly, the tumor interiors. The success of the combination *in vivo* is, however, yet to be tested. We believe that screening drugs by exhaustively addressing the dynamics of the tumor microenvironment at the early stage of pre-clinical trials, will increase the success rates *in vivo* and eventually, in further trials.

**Figure:**
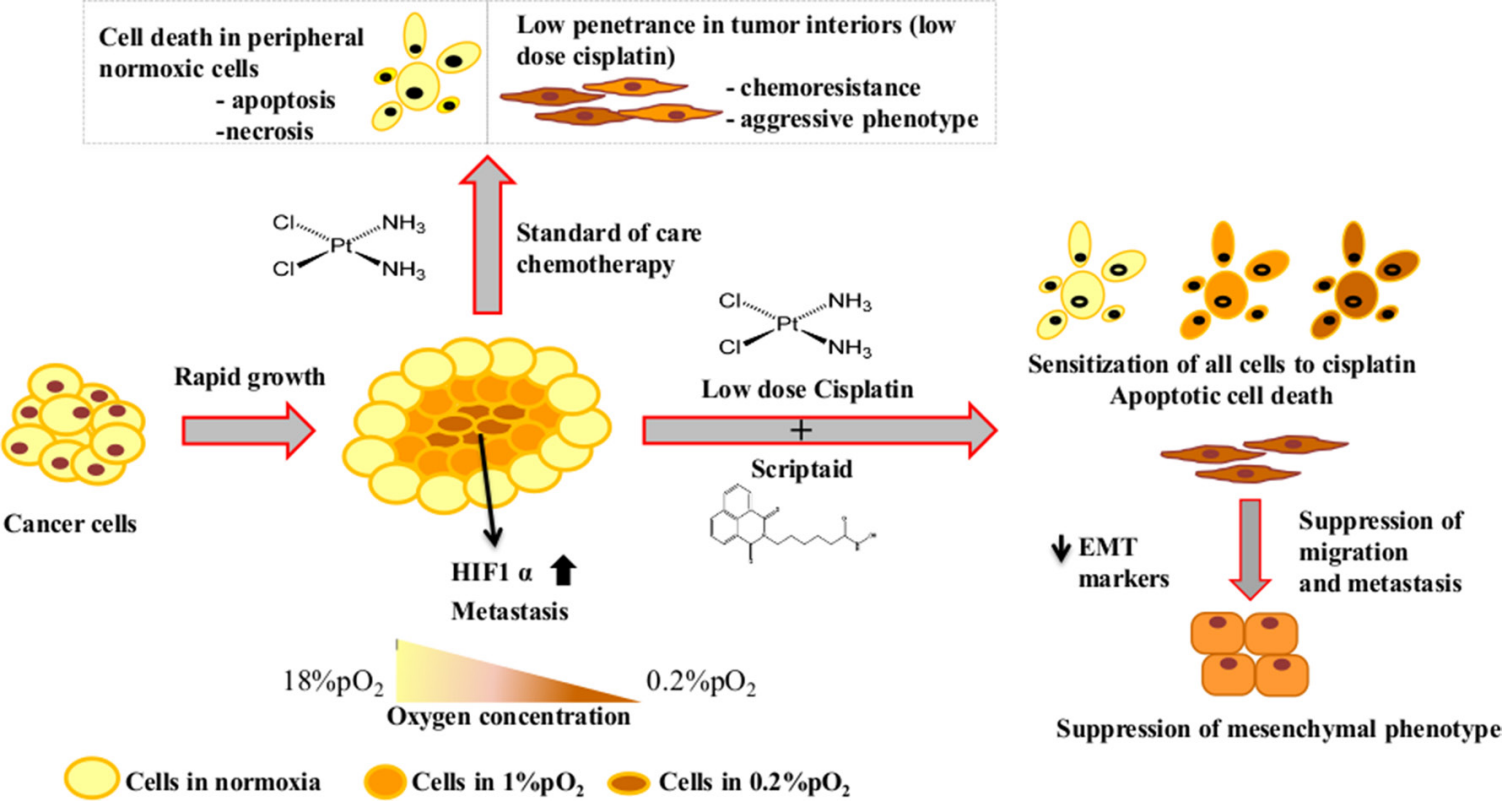
Schematic representation of the effectiveness of combination treatment in lung cancer in conditions mimicking the tumor micro-environment

## MATERIALS AND METHODS

### Cell lines and cell culture

The human lung cancer cell lines- A549 (non-small cell lung cancer) and H460 (large cell lung cancer) were procured from the National Centre for Cell Science (NCCS), Pune and cultured in Dulbecco's Modified Eagle Medium (DMEM, Gibco, USA), supplemented with 10% serum (FBS, Gibco, EU approved). Cells were incubated in a humidified incubator with 5% CO_2_ at 37^°^C. For maintaining *in vitro* hypoxic conditions, cell culture dishes were incubated in Anoxomat chambers (Mart^®^ Microbiology and the Anoxomat^TM^ system) with regulated concentrations of N_2_, CO_2_ and O_2_. Culture conditions with atmospheric oxygen concentration is referred to as normoxia, whereas culture conditions with 1% O_2_ and 0.2% O_2_ is referred to as 1% hypoxia and 0.2% hypoxia respectively.

### Cytotoxicity and viability assays

MTT assay was performed to determine the toxicity of the drugs on lung cancer cell lines. Cells seeded in 96-well plates, at a density of 1 × 10^5^ cells per well, were given the indicated drug treatment-Cisplatin (Sigma, USA) and Scriptaid (Sigma, USA) for 72 hrs. 100 μl of MTT solution (100 μg/ml) was added to each well and after its conversion to a soluble formazan, cell viability was measured by spectrophotometric absorbance at 490 nm. For clonogenic assay, cells were seeded at a density of 1000 cells per well in 6-well plates coated with poly-L-lysine (Sigma, USA). After giving the indicated treatment for 2 hrs, cells were grown in regular growth media for 2 weeks. The resulting colonies were fixed, stained with 0.5% crystal violet and counted using the ImageJ image analysis software.

### Wound healing and migration assay

For wound heal (scratch) assay, 1 × 10^5^ cells per well were seeded to form a monolayer of cells in 24 well plates. A single wound was made in each well using a pipette tip and the indicated treatment given. Images were captured at time 0 and 24 hrs after wound infliction to determine the amount of cell migration. ImageJ software was used to calculate percent migration. In migration assay, cells were seeded at a density of 1 × 10^5^ in transwell inserts in serum-free media. 800 μl of serum-containing media was added in each well and inserts placed on top of each well. Indicated treatments were then given to each well and the plates incubated for 24 hrs. Cells trapped in the membrane of the insert by virtue of the FBS gradient were fixed and stained with Giemsa stain. The images were captured at a magnification of 20× and number of cells counted using the ImageJ image analysis software.

### Cell cycle analysis

Cells seeded in cell culture dishes were given indicated treatments for 48 hrs. The cells were then harvested followed by an overnight fixation in 70% ethanol at −20^°^C. Cells were washed with PBS to remove any trace of ethanol and RNase (Invitrogen, USA) was added to remove RNA from the samples. 40 μg of propidium iodide (Sigma, USA) was then added to each sample and acquired with BD-FACS Calibur. The data so obtained were analyzed by WinMDI software (Version 2.9).

### AnnexinV/PI staining analysis

AnnexinV FITC/PI dual staining is performed to differentiate between apoptotic and necrotic cell death. Cells seeded in cell culture dishes were given the indicated treatments for 48 hrs. The cells were then harvested and suspended in cold PBS. The dual staining using FITC-AnnexinV and PI was done using manufacturer's protocol (#V13242, Invitrogen, USA). The samples were analyzed by flow cytometry, measuring the fluorescence emission at 530 nm and > 575 nm. Flow cytometry analysis was done BD FACS Verse and the experiment performed in three biological replicates. FlowJo software was used for data analysis.

### Plasmid vectors

Two recombinant plasmid vectors were used in the study. The HIF1α mutant coding plasmid- HA-HIF1alpha P402A/P564A-pcDNA3 was gifted by William Kaelin [29] (#18955). The expressed protein has a mutation at amino acid position 402 and 564, which prevents its degradation under normoxic conditions and retains its function. The p53-dominant negative expressing plasmid- pCMV-Neo-Bam-R175H was gifted by Bert Vogelstein [30] (#16436). The mutant p53 protein has a mutation at amino acid position 175. This dominant negative protein dimerizes with the wild-type p53 and prevents its normal functioning.

### Immunofluorescence and confocal microscopy

Cells were cultured in chamber slides and given the indicated treatments. Cells were fixed with 4% paraformaldehyde washed twice with cold PBS and then permeabilized with 0.2% Triton X-100 for 5 mins at room temperature. For immunostaining, cells were incubated with rabbit-anti- γH2AX antibody in recommended dilution overnight at 4^°^C. Alexa Fluor^®^ 488 (Invitrogen, USA) (1:100), prepared in 1% BSA, was added to each well of the chamber slide and incubated for 1 hr. Actin fibers were stained with phalloidin (Cytoskeleton, USA) and nuclei were stained with 4,6-diamidino-2-phenylindole (DAPI) (Sigma, USA). Fluorescent images at the respective wavelengths were first captured on an inverted fluorescence microscope (Olympus IX83, Japan). For high resolution images, confocal images were captured at 60X magnification of the objective (Leica TCS SP5). Image analysis of confocal images was performed using the LAS AF Lite software. CTCF was calculated using ImageJ software.

### RNA isolation and real-time PCR

Cells cultured in petri dishes were given indicated treatments in respective conditions for 24hrs. RNA was isolated using Trizol (Sigma, USA). Total RNA, eluted in 40 μl of nuclease free water, was quantified using Nanodrop (Thermo Scientific, USA). 1μg of total RNA was used for preparing 20 μl of cDNA according to manufacturer's protocol (Revert Aid cDNA synthesis, #K1621). All primers for real-time PCR were prepared using the Primer3 online tool. Real-time PCR was performed in Rotor-Gene Q (Qiagen) by using FIREPol EvaGreen (Solis Biodyne, #08-24-00001) master mix. β-2-microglobulin (B2M) was used as the internal control. Each experiment was performed in three replicates.

### Western blotting

Cells were washed with cold PBS and the cells harvested in 1× lysis buffer. Protein estimation was performed using Bradford assay according to manufacturer's protocol (Biorad, USA #500-0201). 40 μg of each lysate was separated by SDS-PAGE and transferred onto PVDF membrane (MDI, India). The proteins in the membrane were probed with specific antibodies and detected by chemiluminescence (Thermo Scientific, USA).

### Statistical analysis

All *in vitro* experiments were performed at least in three biological replicates for reproducibility of data. Each data point represents at least three technical replicates. Bars in the graph represent standard deviation. Student's *t*-test was used for comparing the difference between two groups and *p* < 0.05 was considered as statistically significant.

### Antibodies used

PARP- full length (#9542), PARP- cleaved (#9541), p53 (#9282), γ-H2AX (#9718), caspase 8 (#9746S), p38MAPK (#9212) and p-p38MAPK (#9211) antibodies were procured from Cell Signaling Technology, USA. Anti-histone H4 (acetylated) (#ab51997) and caspase 3 (#ab32351) antibodies were procured from Abcam, UK. NFκB (#610869) and HIF1α (#610959) was procured from BD Biosciences, USA and β-actin (#NB600-501) from Novus Biologicals, USA.

## SUPPLEMENTARY MATERIALS FIGURES


