# Supplementary material for: Scriptaid overcomes hypoxia-induced cisplatin resistance in both wild-type and mutant p53 lung cancer cells
Source: Oncotarget. 2016 Sep 30;7(44):71841–55. doi: 10.18632/oncotarget.12378 (PMC5342127; doi:10.18632/oncotarget.12378)
Supplement: Supplementary file 1 [file oncotarget-07-71841-s001.pdf]

Scriptaid overcomes hypoxia-induced cisplatin resistance in both wild-type and mutant p53 lung cancer cells

Supplementary Materials

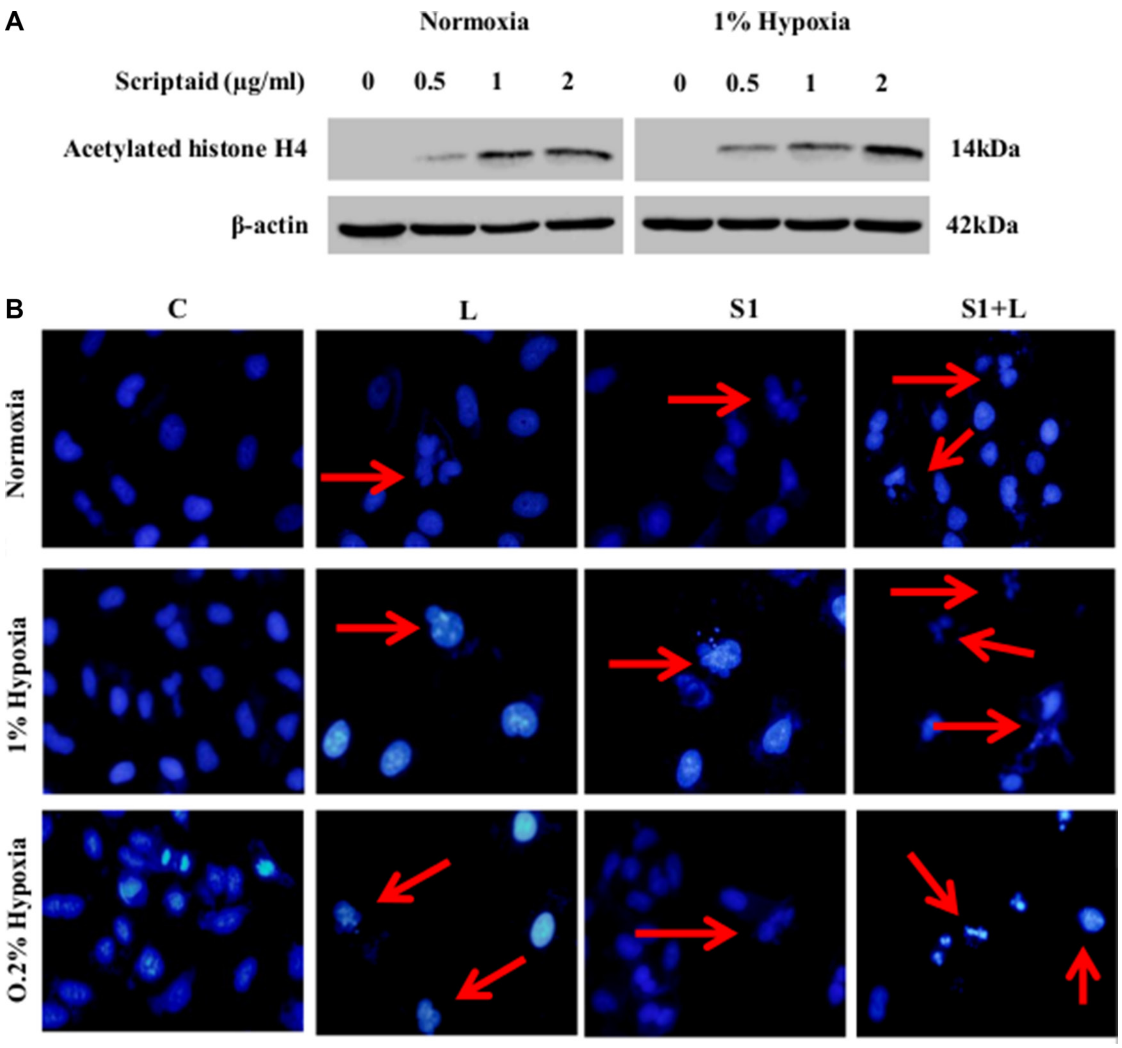

Supplementary Figure S1: (**A**) Western blot analysis showing up overexpression of acetylated histone H4 in response to scriptaid treatment in A549 cell line in a dose-dependent manner in normoxia and hypoxia. (**B**) DAPI stained nuclei showing morphological features of apoptotic cells in response to treatment. Arrows indicate blebs in the nucleus, a hallmark of cells undergoing apoptosis. Combination treated cells showed higher number of apoptosing cells.

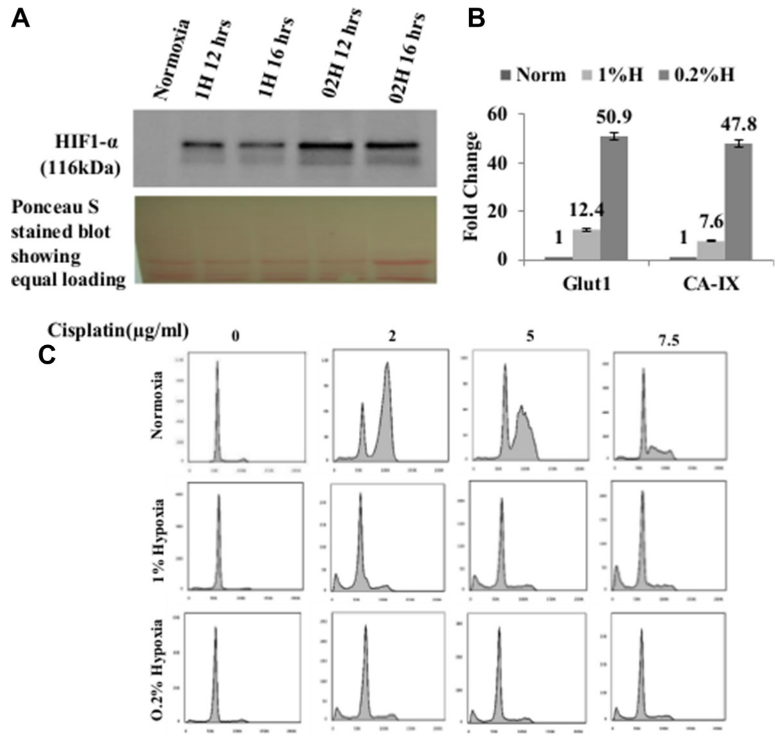

	Cell cycle phase	%population ± standard error			
		Control	2	5	7.5
Normoxia	G_0-G_1	85.8 ± 1.2	18.7 ± 1.32	19.9 ± 1.34	55.7 ± 2.04
	S	6.19 ± 0.66	18.9 ± 0.35	37.7 ± 0.57	24.8 ± 2.5
	G_2-M	6.29 ± 0.18	58.9 ± 1.04	28.9 ± 2.0	12.8 ± 0.53
	Sub-G_0	1.7 ± 0.34	4.5 ± 0.34	8.4 ± 1.58	11.6 ± 0.39
1% Hypoxia	G_0-G_1	83.8 ± 0.29	65.7 ± 1.24	60 ± 1.37	58.8 ± 2.15
	S	2.8 ± 0.32	7.4 ± 1.28	8.5 ± 0.81	8.9 ± 0.99
	G_2-M	4.18 ± 0.51	6.6 ± 0.48	8.9 ± 0.52	7.7 ± 0.31
	Sub-G_0	7.5 ± 0.71	12.6 ± 2.11	17.4 ± 1.89	16.7 ± 2.91
O.2% Hypoxia	G_0-G_1	82.5 ± 1.37	68.2 ± 1.17	68.4 ± 2.01	67.9 ± 1.61
	S	4.4 ± 0.27	7.6 ± 0.18	7.7 ± 0.51	8.7 ± 0.65
	G2-M	5.8 ± 0.57	8.4 ± 0.12	7.8 ± 0.23	7.2 ± 0.53
	Sub-G_0	5.7 ± 1.06	11.8 ± 1.09	9.9 ± 2.24	10 ± 2.09

Supplementary Figure S2: Confirmation of *in vitro* hypoxia by (A) western blot showing stabilization of hypoxia inducible factor (HIF) 1α and up-regulation of HIF1α-regulated genes (B) in A549. (C) Cell cycle analysis by flow cytometry showing G_2-M and S phase arrest of A549 cells in response to cisplatin treatment in normoxia. Arrest was overcome in hypoxia accompanied by increase in G_0-G_1 population.

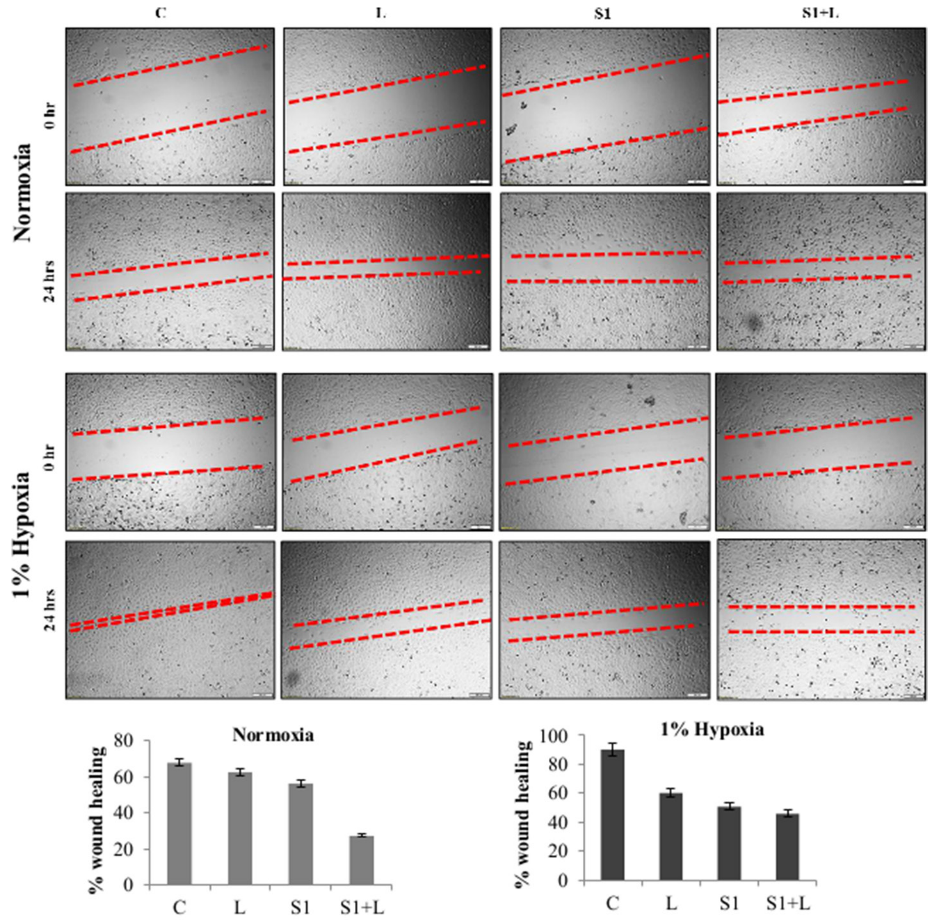

Supplementary Figure S3: Wound healing assay showing inhibition of migration in response to treatment. Compared to normoxia, A549 cells showed aggressive migratory potency under hypoxic condition in untreated controls. The combination treatment was inhibitory to cell migration in both conditions. C = control, L = 2 μg/ml cisplatin, S1 = 1 μg/ml scriptaid. C = control, L = 2 μg/ml cisplatin, S1= 1 μg/ml scriptaid. * indicates $p < 0.05$.

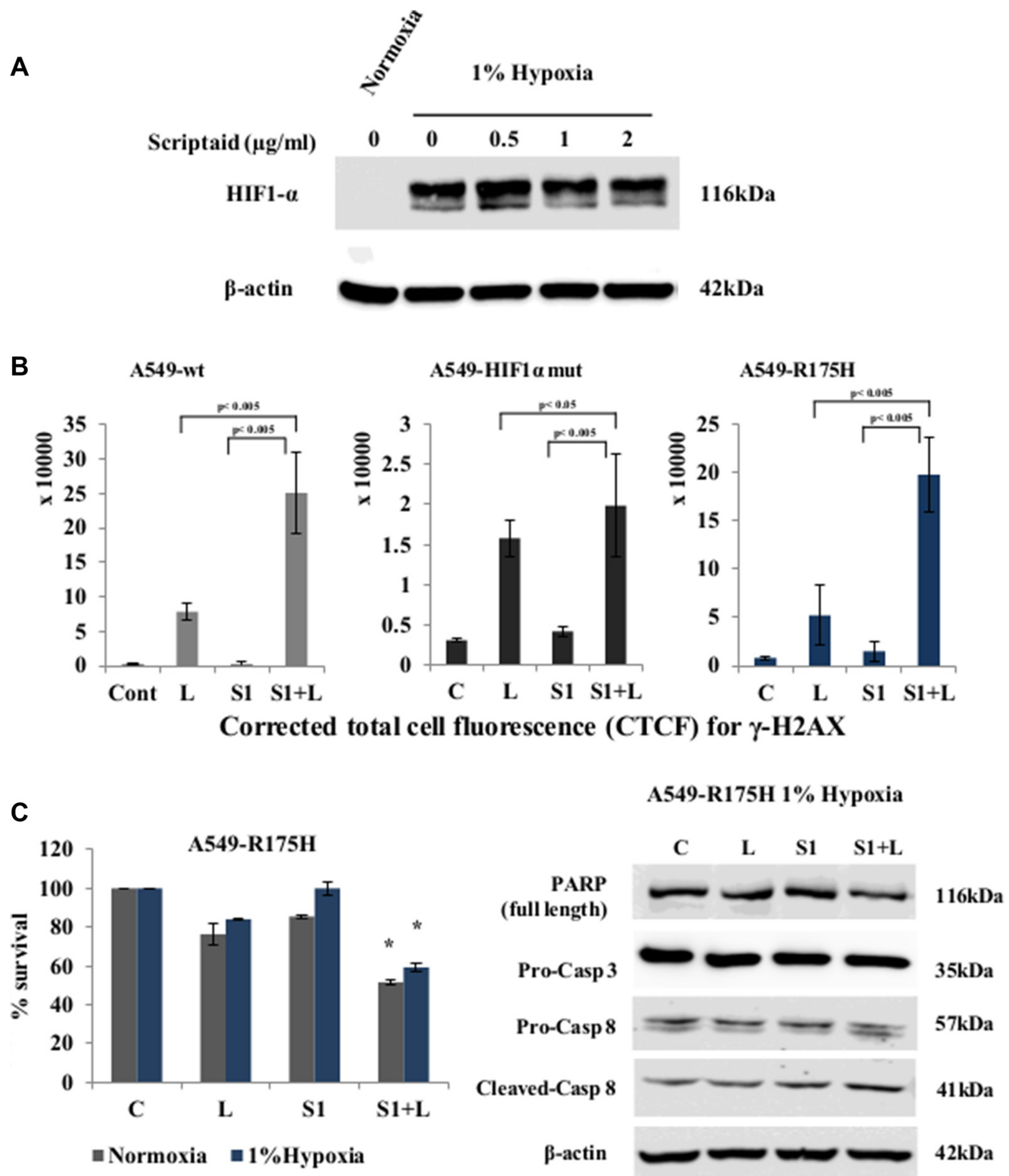

Supplementary Figure S4: (**A**) Western blot for HIF1α shows no significant change in expression after 24 hrs treatment with various concentration of scriptaid in A549 cells. β-actin is used as the loading control. (**B**) CTCF for γ-H2AX was calculated using Image J software; in all 3 cell lines, the combination treatment resulted in significantly more DNA damage than single agent treatment. (**C**) MTT for single and combination in normoxia and hypoxia in A549-R175H cells showing effectiveness of the combination in both conditions; western blot for apoptosis markers in hypoxia for A549-R175H shows increased cell death in combination than that in single agent treatment. C = control, L = 2 µg/ml cisplatin, S1 = 1 µg/ml scriptaid. * indicates $p < 0.05$.
